# Phylogeny and Underground Adaptation of Eulipotyphla Revealed by Whole Genome Comparison Phylogeny and Adaptation of Eulipotyphla

**DOI:** 10.3390/genes17020142

**Published:** 2026-01-28

**Authors:** Hanbing Zhang, Xi Liu, Zhengyu Lin, Li Li, Mingyue Gao, Jialin Sun, Ruihan Li, Hongliang Lu, Kexin Li

**Affiliations:** 1 College of Life and Environmental Sciences, Hangzhou Normal University, Hangzhou 311121, China; 2023210304025@stu.hznu.edu.cn; 2 State Key Laboratory of Grassland Agro-Ecosystem, Institute of Innovation Ecology, College of Ecology, Lanzhou University, Lanzhou 730000, China; liuxi@biols.ac.cn (X.L.); lzhengyu2023@lzu.edu.cn (Z.L.); gmingyue2024@lzu.edu.cn (M.G.); sunjl2023@lzu.edu.cn (J.S.); lruihan2024@lzu.edu.cn (R.L.); 3 Institute of Zoology, Chinese Academy of Sciences, University of Chinese Academy of Sciences, No. 5 Beichen West Road, Chaoyang District, Beijing 100101, China; 4 Institute of Grassland, Xinjiang Academy of Animal Science, Urumqi 830000, China; lily@163.com

**Keywords:** genomics, Eulipotyphla, ecological adaptation, phylogeny, gene flow

## Abstract

**Background:** Phylogenetic relationships within Eulipotyphla have long been debated due to their complex evolutionary history and the frequent inconsistency among phylogenetic trees inferred from different data sources. This order comprises both above-ground and subterranean mammals, providing an opportunity to investigate their adaptation to hypoxic, hypercapnic, and dark environments. **Methods:** In this study, we reconstructed the phylogeny of Eulipotyphla based on whole-genome comparisons and explored the causes of phylogenetic incongruence as well as the genetic basis of underground adaptation. We analyzed the genomes of ten species, including four above-ground species and six subterranean species. We also identified homologous coding sequences through whole-genome alignment and inferred phylogenetic trees based on genome-wide windows of 1000 bases. Divergence times among major lineages were estimated using MCMCtree, and the causes of inconsistent tree topologies were examined using QuIBL to distinguish incomplete lineage sorting from introgression. Finally, we designated the six subterranean species as foreground branches and applied branch-site models to identify genes under positive and negative selection. **Results:** Whole-genome analyses recovered a clear clustering pattern, in which the six subterranean species formed a monophyletic group, whereas the four above-ground species clustered into a distinct clade. Divergence time estimation suggested that the split between above-ground and subterranean lineages occurred approximately 53.51 to 68.78 million years ago. Gene tree analyses revealed substantial variation in tree topologies at several internal nodes, and QuIBL results indicated that introgression contributed to phylogenetic discordance in addition to incomplete lineage sorting. Positive selection analyses identified genes associated with heart regulation, blood circulation, oxidative stress response, and erythrocyte differentiation, while negatively selected genes were linked to cardiac septum and chamber development. **Conclusions:** These results clarify the phylogenetic relationships within Eulipotyphla and provide insights into the genomic basis of adaptation to underground environments.

## 1. Introduction

Phylogeny describes the evolutionary history and relationships of a group of species, tracing lines of descent among organisms. Understanding phylogenetic relationships is essential for elucidating the processes that generate biodiversity, morphological disparity and ecological adaptation across taxa [1,2]. Modern phylogenetic trees are typically inferred from multiple sources of evidence, including morphological characters, mitochondrial and nuclear markers, and increasingly whole-genome datasets [3,4]. However, even genome-scale analyses frequently recover conflicting topologies, reflecting both methodological differences and genuine heterogeneity among loci, and thus complicating efforts to infer a single, fully resolved species tree [5,6,7]. These pervasive inconsistencies highlight the need for approaches that explicitly integrate genome-wide information and model the processes generating gene-tree discordance in order to obtain a more reliable picture of evolutionary history [8,9,10].

Gene-tree conflicts can arise from several biological processes, most notably incomplete lineage sorting (ILS) and introgression, but also horizontal gene transfer, natural selection and convergent evolution [11,12]. Gene flow in particular can lead to genetic homogenization between populations or species, obscuring true relationships and producing topological discordance among loci [13,14]. ILS likewise leaves a characteristic genomic signature because ancestral polymorphisms are stochastically sorted among descendant lineages, especially during rapid radiations, leading to deep coalescence and widespread conflict between gene trees and the underlying species tree [5,15,16]. Distinguishing ILS from introgression is therefore central to interpreting phylogenomic datasets. In three-taxon subsets, the distribution of internal branch lengths provides one diagnostic criterion: ILS tends to produce longer internal branches, whereas recent introgression yields a higher proportion of short internal branches [9,17,18]. Genome-wide sequencing, together with explicit statistical frameworks such as phylogenetic networks, D-statistics and QuIBL, now enables researchers to quantify these processes and to identify the contributions of ILS and introgression to phylogenetic incongruence across clades [11,19,20].

**Figure 1:**
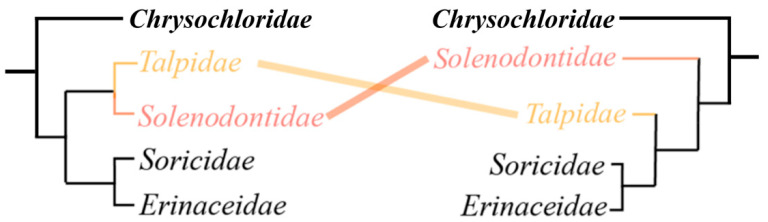
**Species tree differences between Talpidae and Solenodontidae.** The branches highlighted in red and yellow in the figure indicate phylogenetic relationships that are controversial.

Eulipotyphla, also known as Laurasian insectivores, is an order of small mammals that was historically included in the now-defunct Insectivora [21,22]. The group comprises four families—Soricidae (shrews), Erinaceidae (hedgehogs and gymnures), Talpidae (moles and desmans) and Solenodontidae (solenodons)—that exhibit striking diversity in ecology and morphology, yet their interrelationships remain contentious despite extensive molecular and morphological work [23,24]. Most eulipotyphlans are small, insectivorous mammals that rely heavily on olfaction and tactile cues, while vision is often reduced, particularly in subterranean and nocturnal taxa [25]. Previous phylogenetic studies of Eulipotyphla have mainly used mitochondrial genomes, a handful of nuclear loci or ultraconserved elements (UCEs), and have yielded conflicting topologies among families and within Talpidae [26,27]. For example, maximum-likelihood analyses of concatenated nuclear genes have variously recovered Soricidae plus Erinaceidae or Talpidae plus Solenodontidae as sister clades, depending on marker choice and analytical framework [23,28]. Coalescent species-tree approaches based on UCEs have proposed alternative arrangements of these families (Figure 1), underscoring persistent uncertainty in the deep phylogeny of the order. Beyond their systematic interest, Eulipotyphla spans terrestrial, semi-aquatic and subterranean habitats, making the group an excellent model for investigating convergent adaptations to hypoxia, hypercapnia, mechanical constraints of burrowing and life in constant darkness [29,30].

In this study, we use whole-genome comparisons to clarify the contentious phylogeny of Eulipotyphla and to link evolutionary history with ecological transitions between surface and subterranean life [31]. Our dataset comprises ten species, including four primarily terrestrial taxa (*Erinaceus europaeus*, *Crocidura indochinensis*, *Sorex araneus* and *Cryptotis parvus*) and six subterranean or fossorial species (*Uropsilus gracilis*, *Galemys pyrenaicus*, *Condylura cristata*, *Scalopus aquaticus*, *Talpa occidentalis* and *Solenodon paradoxus*). By analysing thousands of genome-wide windows and modelling discordance among gene trees, we aim to obtain a robust species tree, quantify the contributions of ILS and introgression to phylogenetic conflict, and estimate divergence times among major lineages. We further test for signatures of positive and purifying selection associated with subterranean branches to identify candidate genes and pathways involved in adaptation to hypoxia, hypercapnia and life in darkness. Together, these analyses provide a genomic framework for understanding the evolution of Eulipotyphla and generate hypotheses about the molecular mechanisms underlying convergent subterranean phenotypes [30,32].

## 2. Materials and Methods

The genomes of nine species were aligned to the reference genome, *T. occidentalis*, using MAFFT [33], a reliable and accurate tool for multiple sequence alignment that is particularly effective in identifying conserved regions across genomic sequences. The alignment results were then subjected to a stop codon filtering process using the Perl script filter.pl, producing a filtered alignment file (filter.fa).

Gene trees were constructed using IQ-TREE (version 1.6.12) to analyze the evolutionary relationships among species [34,35]. The filtered alignment (A4GALT.filter.fa) was used as input for IQ-TREE with 50 runs (iqtree-s A4GALT.filter.fa--runs 50) to ensure robust phylogenetic inference. Foreground branches were labeled using the HyPhy aBSREL method (hyphy absrel--alignment.filter--tree /data/01/user246/byt/shutu/01/tree.tree--output.out), with six subterranean species and an outgroup designated as the foreground branches.

Additionally, phylogenetic analyses were enhanced with MCMCtree [36,37], implemented in PAML version 4.9j, focusing on synonymous sites within coding sequences where nucleotide substitutions do not alter the encoded amino acid. These synonymous sites, extracted from homologous sequences, are considered evolutionarily neutral and less affected by selective pressures, making them ideal for time-calibrated evolutionary analyses. To increase the resolution, the genome was divided into windows of 1000 bases each, allowing for a detailed examination of species’ evolutionary relationships.

The combined use of IQ-TREE and MCMCtree provided a robust framework for understanding phylogenetic relationships and exploring adaptive evolution in subterranean species. To explore the causes of incongruent tree topologies observed in the phylogenetic analysis, QuIBL was used [19]. QuIBL provides insights into factors such as incomplete lineage sorting, hybridization, and introgression that may contribute to conflicts among tree topologies. The analysis was conducted with *T. occidentalis* designated as the outgroup, providing a rooted framework for evaluating phylogenetic relationships.

The branch-site model in Hyhy packge was applied to test for evidence of both positive and purifying selection, with six subterranean species designated as foreground branches [38,39]. This approach enabled the identification of genes potentially involved in adaptive evolution specific to these lineages. Genes with a *p*-value < 0.05 and an omega value (ω, representing the ratio of nonsynonymous to synonymous substitutions) greater than 1 were identified as candidate genes under positive selection. A custom Python 3.7 script was used to filter and extract these positively selected genes (PSGs), which were further analyzed to explore their potential roles in adaptation to subterranean environments. The final results were visualized using bubble enrichment plots, highlighting the pathways and functions enriched among the PSGs, providing insights into the molecular mechanisms underlying their unique adaptations [40].

Positive selection genes (PSGs) were identified using a custom Python script, filtering for genes with a *p*-value < 0.05 and ω (omega) > 1, indicating strong signals of positive selection. The final results were visualized as bubble enrichment plots, highlighting the enriched pathways and functions in the positively selected genes.

Gene Ontology (GO) analysis was performed to classify and annotate the candidate genes [41]. The analysis utilized Metascape (https://metascape.org/gp/ (accessed on 16 January 2025)), an integrated tool for functional annotation and pathway enrichment analysis, to explore the biological processes, molecular functions, and cellular components associated with the candidate genes. The ten species were classified into two ecological groups—above-ground and subterranean. Among the subterranean species, *U. gracilis*, *G. pyrenaicus*, *C. cristata*, *S. aquaticus*, *T. occidentalis*, and *S. paradoxus*, positively selected genes were identified. These genes provide critical insights into the genetic adaptations that have enabled these species to thrive in underground habitats, highlighting the evolutionary pressures unique to their ecological niche.

## 3. Results

### 3.1. The Sample Collection

The genomes and corresponding annotated files for ten species within the Eulipotyphla order were retrieved from the NCBI database (Table 1). These species were categorized into two ecological groups: four terrestrial species (*E. europaeus*, *C. indochinensis*, *S. araneus*, and *C. parvus)* and six subterranean species (*U. gracilis*, *G. pyrenaicus*, *C. cristata*, *S. aquaticus*, *T. occidentalis*, and *S. paradoxus*). The inclusion of both ecological groups enabled comprehensive comparative analyses to explore their evolutionary adaptations.

The genomic assemblies exhibited varying quality metrics, as reflected in their contig N50 values and GC content percentages. The terrestrial species exhibited contig N50 values ranging from 4.9 kb (*C. indochinensis*) to 4 Mb (*S. araneus*), while the subterranean species ranged from 43.6 kb (*U. gracilis*) to 2.6 Mb (*T. occidentalis*). GC content varied between 40% and 43% across all species, with no significant differences between the two ecological groups (Table 1).

To investigate their evolutionary relationships, homologous sequences shared among these species were identified through whole-genome alignment. The alignment process allowed the identification of conserved regions, providing the basis for downstream phylogenetic and comparative genomic analyses. These homologous sequences were critical in uncovering the genetic basis of ecological adaptations and evolutionary divergence within the Eulipotyphla.

### 3.2. Constructed Phylogenetic Tree of Eulipotyphla

**Figure 2:**
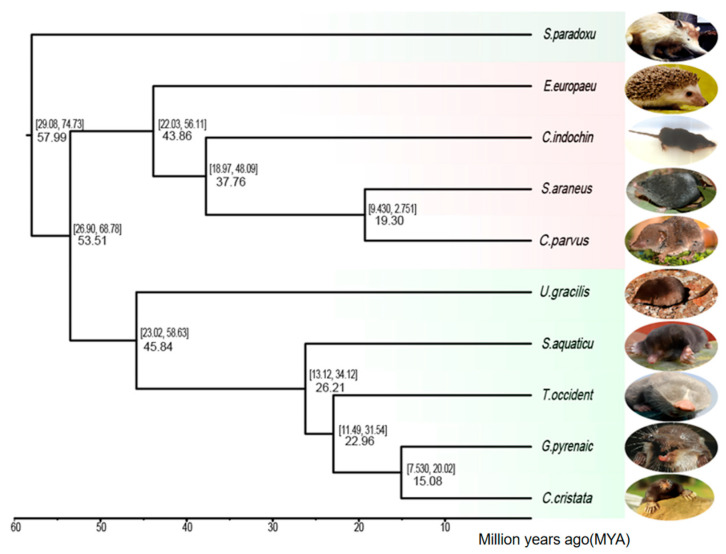
Phylogeny of ten species of *Eulipotyphla*.

Eulipotyphla [21] is a group of small mammals with phylogenetic relationships that have long been a subject of debate due to their complex evolutionary history [6,42]. In this study, we reconstructed a robust phylogenetic tree to clarify the evolutionary relationships within this group. Using whole-genome comparisons, we established the phylogenetic relationships among species in Eulipotyphla with high resolution (Figure 2).

The reconstructed phylogenetic tree was based on 1267 orthologous genes identified across the ten species. This extensive dataset provided the foundation for our high-resolution analysis. Phylogenetic trees were constructed based on positions in coding sequences where nucleotide substitutions do not alter the encoded amino acid. These sites were extracted from the homologous sequences as they are often considered neutral and less likely to be influenced by selection, making them suitable for phylogenetic inference. For this study, the genome was divided into 6424 windows, each of 1000 bases, to ensure a high resolution in the phylogenetic analysis. The tree revealed a clear clustering pattern: six subterranean species consistently formed a monophyletic group, indicating a shared evolutionary trajectory likely influenced by their adaptations to underground habitats. In contrast, the four above-ground species grouped together into a distinct clade, reflecting their divergent evolutionary paths and ecological niches [43].

Furthermore, divergence time estimation revealed that the split between the ground-dwelling and subterranean lineages occurred approximately 53.51 to 68.78 million years ago (Mya), suggesting that these groups have been evolving independently since the late Cretaceous to early Paleocene period. These results provide new insights into the evolutionary history and adaptive divergence of Eulipotyphla, highlighting the role of ecological specialization in shaping their phylogenetic relationships [26].

### 3.3. Cause for the Inconsistency of Phylogenetic Trees

**Figure 3:**
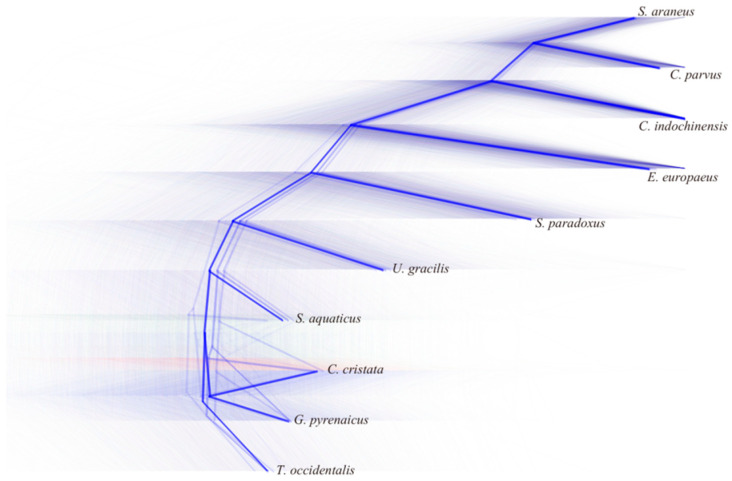
**The phylogenetic relationships constructed by DensiTree.** The shaded areas in different colors in the figure represent different topological structures.

**Figure 4:**
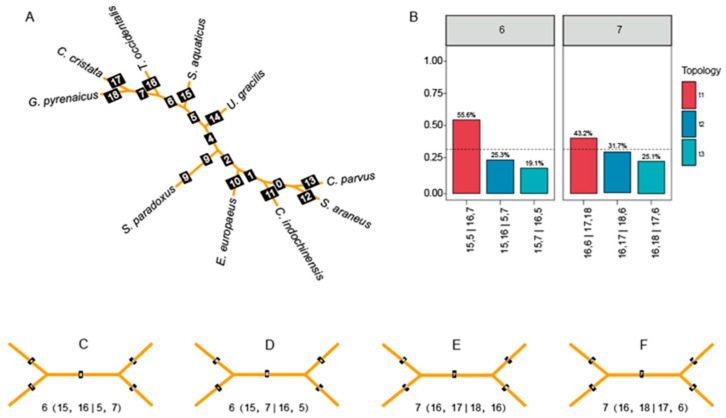
The discordance between gene tree and species phylogenetic tree. (**A**) The dominant topology takes up 75.2% of 100 kb neutral loci window trees. The number in black blocks represents each node. (**B**) Frequency of three bipartitions induced in branches 6 and 7 of ASTRAL species trees using the 100 kb neutral site sliding window; headers 6 and 7 correspond to the branch identity in (**A**). The x axis represents the exact definition of each quartet topology. The main topologies are in red, and the other two alternative topologies are in blue and green. The dashed lines indicate the 1/3 threshold. (**C**,**D**) The discordance tree structures in node 6 correspond to the blue bar and green bar in (**B**) (**Left**), respectively. (**E**,**F**) The discordance tree structures in node 7 correspond to the blue bar and green bar in (**B**) (**Right**), respectively.

The gene trees, constructed from shared homologous coding regions, are presented in Figure 3 and Figure 4. The observed gene tree topologies exhibit significant variation across coding regions [44], highlighting phylogenetic discordances, particularly at nodes 6 and 7 (Figure 4A). The phylogeny was rooted using *Chrysochloris asiatica* an outgroup, enabling the direction of evolution to be determined.

At node 6, the most prevalent topology, (15,5|16,7), accounted for 55.6% of all gene trees, indicating it as the dominant evolutionary relationship in this region. Two alternative topologies were also identified, with (15,16|5,7) representing 25.3% and (15,7|16,5) comprising 19.1% of the gene trees, respectively. These variations suggest differing evolutionary histories among coding regions contributing to the observed discordance [45].

Similarly, at node 7, the primary topology, (16,6|17,18), was observed in 43.2% of all gene trees [46]. Additional topologies, (16,17|18,6) and (16,18|17,6), were identified, representing 31.7% and 25.1% of gene trees, respectively. This variation further reflects the complex phylogenetic relationships among the species, potentially driven by lineage-specific evolutionary events or incomplete lineage sorting.

These results underscore the necessity of integrating multiple gene trees to accurately reconstruct species relationships and identify the evolutionary processes shaping their genomes (Table 2).

## 4. The Adaptation for Underground in Eulipotyphla

Natural selection analyses were conducted to investigate the adaptive evolution of subterranean Eulipotyphla. Using the six subterranean species—*U. gracilis*, *G. pyrenaicus*, *C. cristata*, *S. aquaticus*, *T. occidentalis*, and *S. paradoxus*—as foreground branches, genes under positive selection were identified. This analysis focused on detecting signatures of purifying selection, which removes deleterious variations, and directional selection, which promotes advantageous alleles, providing insights into the genetic basis of adaptation to subterranean environments [47,48].

**Figure 5:**
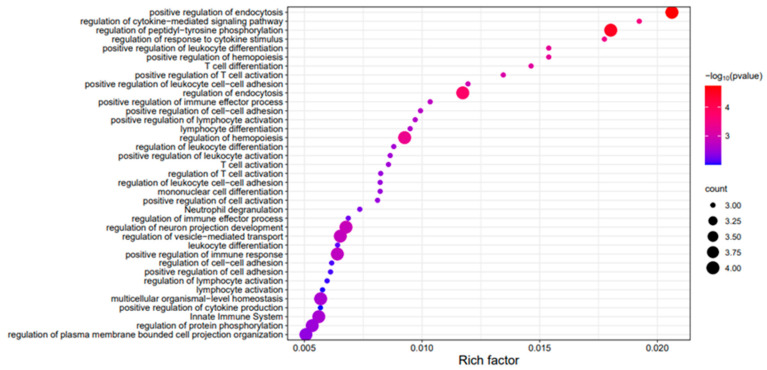
Gene ontology enrichment of positively and negatively selected genes in Eulipotyphla.

The enrichment analysis of 120 genes under positive selection (Figure 5) revealed significant associations with key biological processes and pathways, including cytokine-cytokine receptor interaction, fibrinolysis, response to viruses, regulation of chromatin organization, and defense response regulation. These enriched pathways suggest that adaptations to subterranean environments have driven critical changes in immune responses, cellular signaling, and DNA repair mechanisms. The enriched “defense response” mainly reflects immune defense, supported by immune pathways and T cell–related genes, indicating a role of immune regulation in subterranean adaptation. Specifically, genes like *Tnfrsf1a*, *Il12rb2*, and *Ptprc*, among others, play pivotal roles in these processes. The findings underscore the molecular mechanisms underlying the evolution of subterranean Eulipotyphla, highlighting the influence of natural selection on traits essential for life underground, such as enhanced immune function and stress response regulation, tailored to their unique ecological pressures [49].

The findings provide insights into the molecular mechanisms underlying the evolution of subterranean Eulipotyphla, emphasizing the role of natural selection in shaping traits essential for their specialized lifestyle.

## 5. Discussion

By integrating whole-genome data from ten eulipotyphlan species spanning terrestrial and subterranean ecologies, our study reconstructs a high-resolution phylogeny for the order and links tree topology with signatures of genomic adaptation. Comparisons between four above-ground species (*E. europaeus, C. indochinensis*, *S. araneus* and *C. parvus*) and six subterranean or fossorial species (*U. gracilis*, *G. pyrenaicus*, *C. cristata*, *S. aquaticus*, *T. occidentalis* and *S. paradoxus*) allow us to evaluate whether ecological shifts to underground life are associated with convergent genomic changes and to reassess long-standing hypotheses about relationships within Eulipotyphla [23,26,29].

Although assembly contiguity varied among species—particularly within subterranean lineages—the relatively homogeneous GC content and the recovery of more than one thousand orthologous coding genes across all taxa indicate that current genomes are sufficiently complete for robust comparative and phylogenomic analyses (Table 1). Nonetheless, future chromosome-level assemblies will further refine inferences about structural variation and regulatory evolution associated with subterranean adaptation.

Genome-wide homologous sequences yielded a well-supported species tree in which the six subterranean taxa form a monophyletic clade, whereas the four terrestrial species cluster in a separate lineage, consistent with a major ecological split within Eulipotyphla [24,50]. Our divergence-time estimates place the separation of subterranean and surface lineages in the late Cretaceous to early Paleocene, broadly overlapping with previous molecular-clock analyses for the group and with a period of substantial climatic and tectonic change that reshaped northern hemisphere habitats [21,23]. This temporal framework suggests that ecological opportunities and constraints associated with post–Cretaceous environments may have promoted repeated transitions to fossoriality and the consolidation of an underground-adapted clade.

Despite the strongly supported species tree, individual gene trees showed substantial topological heterogeneity, particularly at nodes 6 and 7, where alternative resolutions each accounted for 20–30% of loci. Such patterns of pervasive discordance are characteristic of rapid radiations in which ILS and episodic introgression both shape genealogies [5,44,45]. Our QuIBL analyses indicate that, for several key triplets, models including introgression fit the internal-branch length distribution better than ILS-only models, implicating historical gene flow among early-diverging lineages as an additional driver of conflict, as has been reported in other mammalian and plant clades [19,20,51].

Our branch-site selection tests identified sets of positively selected genes enriched for functions related to cardiac development, muscle contraction, erythrocyte differentiation, vasculature development and responses to oxidative and immune stress. These processes are plausibly linked to the chronic hypoxia, hypercapnia and mechanical demands of burrowing in subterranean environments, where efficient oxygen transport and cardiovascular remodeling are essential [30,47,48]. Similar enrichment of hypoxia- and stress-response pathways has been reported in other subterranean and high-altitude mammals, suggesting convergent molecular solutions to life under low oxygen and high CO_2_ [25,52]. The overlap between our candidate genes and pathways highlighted in these systems strengthens the inference that natural selection has repeatedly targeted cardiopulmonary and stress-response networks during the evolution of subterranean Eulipotyphla.

Taken together, our results refine the deep phylogeny and divergence times of Eulipotyphla, quantify the contributions of ILS and introgression to gene-tree conflict, and identify candidate genomic targets of selection associated with subterranean life. By combining whole-genome phylogenetics, network-based tests of gene flow and branch-site selection analyses, this study illustrates how integrative genomic approaches can disentangle complex evolutionary histories and link them to ecological transitions in non-model mammals [4,23]. Future work incorporating chromosome-level assemblies, regulatory and transcriptomic data, and broader taxon sampling will further illuminate how changes in gene regulation, structural variation and genome architecture contribute to the repeated evolution of underground adaptation in this ecologically diverse order.

## Figures and Tables

**Table 1:** The genome of 10 species in Euipotyphla.

Scientific Name	Contig N50	GC Percent	GenBank/RefSeq	Assembly	Ecology
*Galemys pyrenaicus*	236.8 kb	40	GCA_019455555.1	Gpyr_1.0	Subterranean
*Condylura cristata*	46.2 kb	41.5	GCF_000260355.1	ConCri1.0	Subterranean
*Talpa occidentalis*	2.6 Mb	42	GCF_014898055.3	MPIMG_talOcc4v2.1	Subterranean
*Scalopus aquaticus*	72.4 kb	42	GCA_004024925.1	ScaAqu_v1_BIUU	Subterranean
*Solenodon paradoxus*	236.8 kb	40	GCA_004363575.1	SolPar_v1_BIUU	Subterranean
*Erinaceus europaeus*	999.9 kb	42	GCF_950295315.1	mEriEur2.1	Subterranean
*Crocidura indochinensis*	4.9 kb	40	GCA_004027635.1	CroInd_v1_BIUU	Above-ground
*Sorex araneus*	4 Mb	43	GCF_027595985.1	mSorAra2.pri	Above-ground
*Cryptotis parvus*	112.3 kb	43	GCA_021461705.1	Cryptotis parva assembly 1.0	Above-ground
*Uropsilus gracilis*	43.6 kb	41	GCA_004024945.1	UroGra_v1_BIUU	Above-ground

**Table 2:** Discrimination of introgression from incomplete lineage sorting by QuIBL.

Triplet	Outgroup	C1	C2	Mixprop1	Mixprop2	Lambda2dist	Lambda1dist	BIC2Dist	BIC1Dist	Count
18_16_15	18	0	1.892047	0.341483	0.658517	0.003866	0.007163	−148,339	−145,320	18,448
18_16_15	16	0	2.010333	0.373425	0.626575	0.00294	0.005501	−97,506.6	−95,993.1	11,421
18_16_15	15	0	2.126256	0.294418	0.705582	0.003335	0.006724	−182,490	−177,593	22,189
18_16_17	18	0	2.041335	0.333604	0.666396	0.003567	0.006889	−152,314	−149,030	18,734
18_16_17	16	0	1.886331	0.350917	0.649083	0.003707	0.006807	−152,068	−149,272	18,708
18_16_17	17	0	2.11837	0.337182	0.662818	0.003343	0.006617	−120,056	−117,446	14,616

## Data Availability

No new data were created or analyzed in this study.
